# Enablers and Barriers Related to Preconception Physical Activity: Insights from Women of Reproductive Age Using Mixed Methods

**DOI:** 10.3390/nu15234939

**Published:** 2023-11-28

**Authors:** Pragya Kandel, Siew Lim, Michelle Dever, Prabhat Lamichhane, Helen Skouteris, Sinead Currie, Briony Hill

**Affiliations:** 1 Health and Social Care Unit, Monash University, Melbourne, VIC 3004, Australia; pragya.kandel1@monash.edu (P.K.); michelle.dever@monash.edu (M.D.); helen.skouteris@monash.edu (H.S.); 2 Health Systems and Equity, Eastern Health Clinical School, Monash University, Melbourne, VIC 3004, Australia; siew.lim1@monash.edu; 3 Department of Public Health, School of Psychology and Public Health, La Trobe University, Melbourne, VIC 3086, Australia; p.lamichhane@latrobe.edu.au; 4 Warwick Business School, University of Warwick, Coventry CV4 7AL, UK; 5 Division of Psychology, University of Stirling, Stirling FK9 4LA, UK; sinead.currie@stir.ac.uk

**Keywords:** preconception, PA, behaviour change, women of reproductive age, mixed method, enablers, barriers

## Abstract

Engaging in regular preconception physical activity (PA) is associated with benefits, including improved cardiovascular health and mental well-being. However, most women do not meet PA recommendations in the preconception period. This study aimed to investigate enablers and barriers related to PA in preconception women using a sequential mixed method design. An online survey was followed by Zoom interviews with women of reproductive age (aged 18–45 years). A weaving approach and the Capability Opportunity Motivation Behaviour (COM-B) model were used to integrate and present the data. Seven hundred and eighty-eight non-pregnant women from Australia, India, and the US completed the quantitative survey, and 13 Australian-based women participated in a qualitative interview. Physical activity levels were associated with having social support, a desire to improve body image, and becoming a healthier person. Women encountered barriers such as misconceptions about PA, competing priorities, financial constraints, and a lack of accessibility. Enablers for participation in PA included knowledge of its importance, a desire to be healthier, weight loss, social support, and having goals. The multifaceted and intricate nature of enablers and barriers for preconception PA lays the groundwork for developing tailored interventions and policies aimed at promoting preconception PA among women.

## 1. Introduction

The preconception period is often broadly defined as the time before conception, regardless of a woman’s intention for pregnancy [1]. Engaging in a range of health behaviors before pregnancy, such as participating in appropriate physical activity (PA) and maintaining a healthy diet, can offer numerous benefits to both women and their future children [2]. However, to optimize these benefits, women should be engaging in these health behaviours for months, or even years, before conception [2]. This preconception phase holds significant importance with wide-ranging implications for the health of both women and their children throughout their lives [2]. Engaging in regular PA prior to conception has been linked to various benefits, including improved cardiorespiratory fitness [3], reduced risk of developing gestational diabetes mellitus [4,5] and preeclampsia [6], weight management [7], and improved mental well-being [8], all of which contribute to creating an optimal environment for conception and gestation [2]. Moreover, preconception PA behaviours can have long-lasting effects, potentially influencing not only the immediate future pregnancy but also the health trajectories of future generations [2]. In fact, participation in preconception PA is a significant predictor of continued PA during pregnancy [9,10]. Therefore, the preconception period represents a critical window of opportunity for behaviour change [11].

Despite the benefits of being physically active before conception, research indicates that preconception PA behaviour is often suboptimal among women of reproductive age [10,12,13,14]. Notably, a report published by the Australian government revealed that 78% of women of reproductive age fail to meet the national guidelines, which recommend being active on most, preferably all days, with at least 150 min of moderate to vigorous activity per week [12]. A 2008 Pregnancy Risk Assessment Monitoring System (PRAMS) study found that only 13% of women in the US reported engaging in 30 or more minutes of PA on five or more days per week before pregnancy [15]. Another cross-sectional Canadian study of women planning to conceive found that more than half (57.7%) did not meet the recommended PA [16]. Furthermore, despite contrary belief, data from a study involving 12,391 Australian women of reproductive age, both with and without pregnancy intentions, revealed that they do not necessarily change their behaviour before pregnancy [17]. This gap between the established benefits and the low engagement in PA highlights the need for a comprehensive understanding of the factors that influence women’s decisions regarding participation in preconception PA.

While research on preconception health behaviours is notably scarce, a significant gap exists, particularly in the area of PA [18,19,20]. Existing studies have primarily focused on women who are planning pregnancy [21] and predominantly identified knowledge as a factor that both enables and hinders preconception PA [19]. Greater knowledge has been associated with higher participation in PA [22,23]. However, although knowledge is a crucial ingredient for behaviour change, it alone may not be sufficient to increase PA due to other barriers during the preconception and interconception period. It is therefore essential to delve deeper into other potential factors. Barriers to PA for preconception women may coalesce with the distinct challenges women encounter after childbirth, as the interconception period (time between pregnancies) is a key preconception life phase [24]. These challenges include time constraints and reduced energy levels due to the demands of work, childcare, and various other role-related pressures [7]. However, there remains little clear evidence on the enablers and barriers to preconception PA. With the existing research on preconception PA being limited, there is now a need to gain a deeper and more holistic understanding of the reasons why women are not engaging in regular PA, including unpacking the facilitators and barriers that influence women’s engagement in preconception PA behaviours. This is particularly salient given that PA behaviours are part of a complex process influenced by various factors, some of which may or may not be within individual control, such as social, environmental, and commercial determinants of health [25]. Nevertheless, granting women the chance and agency to enhance their PA behaviours prior to pregnancy remains crucial.

Given the limited understanding of preconception PA behaviour change, the aim of this study was to comprehensively investigate enablers and barriers influencing preconception PA for behaviour change in women of reproductive age. To comprehensively address the multifaceted issue of women’s engagement in preconception PA, we require an approach that not only helps us understand the existing enablers and barriers but also delves into the underlying reasons for these enablers and barriers, specifically uncovering women’s perceptions and thoughts regarding them. Therefore, we used a sequential explanatory mixed-method design guided by three domains of the Capability Opportunity Motivation Behaviour (COM-B) model for behaviour change—capability, opportunity and motivation. Using a mixed methods approach provides deeper insights when tackling complex issues [26,27], thereby guiding the development of specific interventions and strategies aimed at improving women’s preconception PA. Mapping findings to the COM-B Model provides a theory-informed framework for the integration of the quantitative and qualitative data.

## 2. Materials and Methods

We conducted a sequential explanatory mixed-method study [26,28]. An online cross-sectional study was conducted first and analyzed. The results were then used to inform the qualitative study, which provided a more comprehensive context to explain and interpret the quantitative findings.

### 2.1. Quantitative Study

#### 2.1.1. Study Population

An online cross-sectional study was conducted with preconception women using Qualtrics [29]. The STROBE checklist was used in reporting this study when applicable (Supplementary Table S1) [30]. Preconception women were defined as women aged 18–45 years who were not currently pregnant, capturing potentially unplanned pregnancies along with planned pregnancies where the time to conception was uncertain, as well as women who had never been pregnant or were interconception (time between pregnancies) [1]. Women who were able to read in English, had access to the internet, and were not pregnant were eligible. A total of 1346 women were recruited using social media (e.g., Facebook, Twitter), word of mouth, snowballing techniques, emails to researchers’ personal and professional networks, university newsletters and via the online recruitment panel databases Cloud Research and Online Research Unit (ORU). Completion of the online survey indicated implied consent.

#### 2.1.2. Measures and Data Collection

*Dependent Variable: PA.* PA level was evaluated using the validated International Physical Activity Questionnaire Short form (IPAQ-SF; [31]). The IPAQ-SF is a self-reported questionnaire that consists of seven questions about the average daily time spent sitting, walking, and engaging in moderate and vigorous PA over the last seven consecutive day periods. Responses to each question were used to calculate the metabolic equivalent of task (MET)-min/week using the IPAQ protocol [32]. Participants were grouped by their MET-mins/week scores: <600 MET-mins/week as low PA, 600–3000 MET-mins/week as moderate PA, and >3000 MET-mins/week as vigorous PA. The test–retest reliability (intra-class correlations range 0.7–0.8), concurrent (median rho = 0.67), and criterion validity (rho = 0.3) of the IPAQ-SF has been examined among people aged 16–69 years of age and was determined to be acceptable [31]. In the current study, there were only 12 observations in the low category; consequently, the low and moderate categories were merged to create a binary dependent variable, “low/moderate”.

*Independent variable: Enablers and Barriers related to PA.* No suitable measures existed to assess the perceived enablers and barriers to preconception PA. Therefore, we developed a new measure, the Preconception Physical Activity Enablers and Barriers Scale (PPEBS). We applied Brancato et al.’s five stages of questionnaire design and testing to develop the new measure—conceptualization, questionnaire design, testing, revision, and data collection [33]. Following those five stages, an initial pool of items was selected based on previous research [19,34] and on consultation with experts. The expert team consisted of a developmental psychologist, exercise scientist, accredited practicing dietitian, health psychologist, public health expert, and consumer. Items were measured using a 5-point Likert scale from 1 (Strongly disagree) to 5 (Strongly agree). The initial draft of the questionnaire items was piloted with 10 Australian women of reproductive age who were not currently pregnant to ensure that questions were clearly understood and the survey length was appropriate. Revisions were made to the items based on the feedback provided in order to improve clarity. A second version of the questionnaire was retested among a convenience sample of different women of reproductive age who were currently not pregnant (*n* = 20) to establish test–retest reliability. Out of 26 items, nine items that were not significantly correlated were dropped, and the final 17-item version of the questionnaire was incorporated into the main study (Supplementary Table S2). For the current analyses, for each item, “strongly agree” and “agree” were combined, while “neither agree nor disagree”, “disagree”, and “strongly disagree” were combined, resulting in a binary variable. These categorizations were selected because uncertainty tends to lean towards disagreement [35].

*Sociodemographic measures:* The following sociodemographic data were collected: age, marital status, household composition, country of residence, educational status, paid employment status, pregnancy plans for the future, smoking habit, drinking habit and Body Mass Index (BMI).

Time-based checks, IP address monitoring, response pattern analysis, and strategically placed negatively worded statements were used to safeguard against “bot” responses, ensuring data integrity.

#### 2.1.3. Sample Size Calculation and Data Analysis

**Figure 1:**
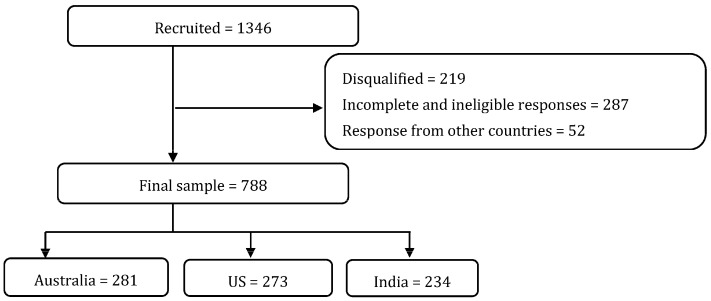
Flow chart of participants.

Based on analogous literature in different populations, to examine the relationship between psychosocial factors/pregnancy planning and PA, a sample size of approximately 483 is required to detect a small-medium effect size (f2 = 0.04) with 80% power at alpha = 0.05. To accommodate the inclusion of participants from diverse countries, thereby enhancing the generalizability of our findings and ensuring a broader representation of populations, we recruited a larger sample. Our final sample size of 1346 women was recruited from 13 different countries, with the majority (93%) coming from the USA, India, and Australia. Observations from 10 countries, which had only a handful of responses each (*n* = 52), were excluded from the analysis to maintain robustness. The final analytical sample consisted of 788 participants, which exceeded our initially calculated sample size. The flow of participants in the study is presented in Figure 1.

Binary logistic regression was performed to examine the association between the dependent variable (PA level; IPAQ-SF) and independent variables (enablers and barriers; PPEBS). First, we examined the bivariate relationship between the level of PA and each statement from PPEBS to obtain the unadjusted odds ratio (OR). Second, we ran the models accounting for potential confounding variables (i.e., age, marital status, household composition, paid employment, country of residence, BMI, smoking habit, drinking habit, and pregnancy planning status, to obtain the adjusted OR (aOR)). These steps were carried out for each individual PPEBS statement. Stata v.15 was used for analysis.

### 2.2. Qualitative Study

#### 2.2.1. Study Population

A total of 13 participants were recruited and participated in semi-structured interviews. These participants were defined as preconception women aged 18–45 years who were currently not pregnant. Women who were able to read and speak in English, had access to the internet, and lived in Australia were eligible. For pragmatic reasons, we chose to interview only women currently residing in Australia, and our quantitative results on PPEBS also showed similar patterns in terms of items between participants across three countries (Australia, India and the US, Supplementary Table S3). Women who were pregnant at the time of the survey were excluded. All participants from the qualitative study provided written informed consent.

#### 2.2.2. Measures and Data Collection

A semi-structured interview guide was developed based on the quantitative findings to gain a holistic understanding of the reasons why women are not practicing regular PA. The guide (Supplementary Table S4) covered questions such as “what do you think are the benefits about the PA?”, “what barriers are for you to be physically active?”, “please tell me about the things that motivate you to be physically active” and basic demographic factors (age, pregnancy history, pregnancy planning status). PK and EM, both with backgrounds in public health and of a similar age to the women, conducted the interviews. All interviews were conducted using Zoom and were audio-recorded and transcribed verbatim. Pseudonyms were used to maintain participant confidentiality. On average, interviews lasted for 36 min (range: 21–52 min) and women were interviewed until data saturation was reached [28].

#### 2.2.3. Qualitative Analysis

Thematic analysis with an inductive approach [36] was used to generate themed groups of enablers and barriers. Data were independently coded by two researchers (PK and MD), and any discrepancies were resolved through discussion. Codes were then consolidated to form themed groups, with the input of both researchers (PK and MD). Inter-coder reliability (ICR) was evaluated using the calculation of percentage agreement/disagreement, which was assessed before consensus was established to assess agreement between coders [37]. Inter-coder reliability ensures that multiple coders consistently interpret and analyze data, enhancing the validity and trustworthiness of research findings, which is suitable when applying enablers and barriers deductively to the COM-B model. Reliability between two coders is considered acceptable if a percentage agreement greater than 60% is achieved [38]. NVivo 1.3 software was used to code and manage the data. Interviews and analysis were conducted in accordance with best-practice guidelines for qualitative research [39].

### 2.3. Mixed Methods Integration and Analysis

Data were integrated at the interpretation and reporting level using a weaving approach and the use of joint display. Weaving allows integration through narrative, which includes writing both the quantitative and qualitative findings together on a theme-by-theme or concept-by-concept basis [40]. Then, we organized the findings in a joint display, where data from both quantitative and qualitative data are presented visually to provide a broader and more comprehensive interpretation of women’s responses to our main variable [41,42]. We used the COM-B model to guide the presentation and conceptualization of our data as it provides a comprehensive theoretical framework for understanding behaviour and behaviour change. Our main variables of interest, i.e., PPEBS, and themed groupings from the qualitative study, were deductively mapped to the COM-B model components via discussion between team members (PK, MD and BH).

## 3. Results

The quantitative and qualitative results are summarized briefly below, with an in-depth analysis provided in the integration of findings section.

### 3.1. Quantitative Study

#### 3.1.1. Participant Characteristics

Participant characteristics are summarized in Table 1. For the total sample, the mean age of participants was 32.1 (SD 7.3) years. Participants mainly reported being married (*n* = 416; 52.7%), belonging to a couple family with children (*n* = 383; 48.7%), holding a Bachelor’s degree (*n* = 302; 8.4%), and being employed (*n* = 563; 72.5%). Additionally, 541 (68.8%) of participants had never smoked cigarettes, and 290 (36.85%) had never consumed alcohol. Three hundred and nine (40.0%) of participants had a BMI in the range of 18.5–24.9 kg/m^2^ (“normal”). The majority of the population (*n* = 492; 62.4%) reported a moderate level of PA, with only a small proportion (*n* = 12; 1.5%) engaging in low levels and 284 (36%) participating in high levels of PA. Horizontal bar charts for each statement for enablers and barriers with all response categories are presented in Supplementary Material (Figure S1).

#### 3.1.2. Associations between PA Levels and Enablers and Barriers Related to PA

Table 2 presents the results of the univariable and multivariable logistic regressions. The univariable analyses showed women who agreed that PA during the preconception period is important, had enough time to be physically active, were willing to be physically active to become a healthy person, were physically active to improve body image, were doing PA, and those who wanted to be a role model for their children were significantly associated with a higher likelihood of high levels of PA. This strong effect was also observed in multivariable analyses (controlling for covariates, including country of residence) for the statements where women agreed that they were doing PA (aOR of 3.73 (95% CI 2.45–5.68)) and had enough time for PA (aOR = 2.1 (95% CI 1.47–2.99)). ‘PA during the preconception is important’ was no longer significant in the multivariable analyses, although the likelihood of a higher level of PA among those women was still higher with 1.29 (95% CI 0.86–1.95). Women who agreed with the statement that they do not have friends’ support for regular PA had a significantly lower likelihood of PA in multivariable analyses (aOR = 0.55 (95% CI 0.33–0.90).

### 3.2. Qualitative Study

#### 3.2.1. Demographic Characteristics

Table 3 presents the demographic characteristics of women in the qualitative study. The age of women ranged from 25 years to 44 years. The majority of women had been pregnant and had planned their previous pregnancies.

The themed groups of enablers and barriers identified using qualitative analysis included knowledge beliefs about consequences, goals, emotions, beliefs about capabilities, social influences, and environmental context and resources. These groups were further categorised into specific enablers and barriers using the COM-B model. The barriers that emerged included misconceptions that only vigorous activity counts as PA, feeling overwhelmed by the information available on social media/internet, lack of confidence, no priority given to oneself, physical exertion, time constraints, lack of social support, financial constraints, and limited accessibility. On the other hand, the enablers for PA included awareness of the importance of preconception PA, understanding PA information available on the internet/social media, belief in the benefits of preconception PA, positive feelings associated with regular PA, having goals, presence of social support, and aspiring to be a role model. These categories are further elaborated in the next section, where both quantitative and qualitative findings are integrated in greater detail.

#### 3.2.2. Integration of Quantitative and Qualitative Study Findings Mapped to the COM-B Model

The findings from both qualitative and quantitative studies were mapped to the three domains of the COM-B model, which are capability, opportunity, and motivation. The joint display of integrated findings by COM-B component, themed groups of enablers and barriers, and identified categories of specific enablers and barriers, including selected statistics and quotes from the quantitative and qualitative studies, is presented in Table 4.

### 3.3. Capability

Knowledge was the capability construct identified for the COM-B model. Several categories were identified that mapped to the knowledge domain. These included awareness/lack of awareness of the importance of preconception PA, accessing information about it (mainly via social media), and misconceptions about PA.

Our quantitative results, as shown in Table 4, highlighted that women had a relatively high degree of awareness of the benefits of preconception PA. These findings were supported by the qualitative discussions, where women further reiterated that PA during the preconception period is very important and beneficial for both women and babies, promoting a healthy pregnancy journey. Women emphasized PA as a pivotal factor benefiting both maternal and fetal health. Women indicated that they thought pregnancy was physically demanding, and they wanted a healthier body in order to give birth successfully.

Despite over three-quarters of women in the quantitative study reporting that they could understand the PA information related to the preconception period available on the internet/social media, qualitative findings highlighted that women often grappled with the overwhelming abundance of information. For example, women told of a surplus of information that made it challenging to work out what was reliable and what was not. This inundating information not only posed a challenge to their understanding but also raised concerns about the potential for misleading content. It was clear from the discussion with women that the presence of misleading and excessive information acted as a barrier to PA. This proliferation of misleading information can lead to confusion and uncertainty among women looking for guidance on preconception PA, potentially discouraging them from engaging in PA altogether. Moreover, the overwhelming volume of information can be intimidating, making it difficult for women to identify credible sources that can genuinely have relevant, accurate information about preconception PA. Women’s difficulty in processing and curating the information available may also explain why awareness of the importance of PA did not translate into higher PA levels in our regression analyses.

Despite a generally good awareness of the importance of PA, findings from the qualitative study showed that misconceptions surrounding preconception PA remain barriers. For example, women held the belief that PA is only of benefit if one participates at the level of vigorous exercise, and this represented a significant barrier to its adoption. When women associate PA with intense workouts, they may not recognize the value of incorporating moderate activities like walking, stretching, or taking children to the park as part of their preconception routine. This misconception might discourage those who are not familiar with high-intensity exercise or those with physical limitations from engaging in PA before conception. This finding may explain why the women surveyed who stated they had enough time to be physically active were more likely to participate in MVPA levels such that recognizing PA comes in many shapes and forms can be an enabler to weaving it into everyday life.

### 3.4. Motivation

Beliefs about consequences (believing in benefits of preconception PA), beliefs about capabilities (lacking confidence and no priority given to oneself), emotions (positive feelings associated with regular PA, physical exertion), and goals were enablers and barriers that mapped to the motivation domain of the COM-B model.

Believing in the benefits of preconception PA acted as a crucial enabler in motivating women to engage in regular PA, fostering their overall health and the health of potential babies in the future. Table 4 shows that quantitatively, participants strongly believed in the positive impact of preconception PA on their own general health and on the health of potential future offspring. Qualitative insights further supported these findings; women consistently described preconception PA as a means to become a healthier person, improve their pregnancy journey and the health of their baby, improve sleep quality, gain more energy for family interactions, bolster mental health, promote a healthy baby, prevent injuries, build physical strength, manage weight, alleviate labor discomfort for a more favorable birthing experience, and even seek respite from the demands of daily life. These findings not only highlight the importance of these beliefs but also emphasize their potential as a powerful lever for targeted interventions.

While emotions were not included in the quantitative survey, qualitative findings reported emotions as a dual role, acting both as an enabler and a barrier that impacted motivation to engage in preconception PA. On the one hand, women expressed profoundly positive emotions and a genuine affection for exercise. These sentiments highlighted the role of positive emotions as powerful enablers, motivating women to embrace PA with enthusiasm. Conversely, emotions such as physical exhaustion and fatigue were identified as significant barriers, as stressed by participants who described the challenges of managing demanding work schedules and daily responsibilities. These qualitative insights revealed the dynamic interplay of emotions as both enablers and barriers, providing valuable perceptions into the complex emotional landscape that shapes behaviour change in this critical preconception period.

Beliefs about capabilities where women lacked confidence and did not give priority to themselves have acted as a barrier to behaviour change to preconception PA. Even though it was not included in the quantitative study, qualitative findings showed that women expressed a notable lack of confidence in their ability to engage in preconception PA. Some women disclosed that unless they felt physically compelled or coerced into action, they tended to craft excuses to avoid engaging in PA. Moreover, the tendency to prioritize others’ needs over one’s own resulted in self-neglect, leaving little room for emphasizing PA. Women talked about the challenges in balancing their PA due to the demanding nature of their caregiving roles, particularly in the context of managing children’s needs. Women described being constantly busy, switching between various responsibilities, which left them with little time or inclination for personal exercise. This has illuminated the significant impact of caregiving responsibilities on individuals’ perceived capabilities to engage in preconception PA. These qualitative findings emphasize the critical need for tailored interventions that address not only the practical challenges posed by caregiving responsibilities but also the psychological barriers that hinder individuals from prioritizing their own health within the preconception care context.

Across both the quantitative and qualitative studies, having goals played a vital role as an enabler for promoting preconception PA, offering supplementary motivation to women to engage in regular PA. In the quantitative study, a significant proportion of women agreed with statements related to their intention to engage in PA with specific objectives in mind. The qualitative study offered a further understanding of these goals, as participants articulated aspirations of becoming healthy, engaging in regular PA, losing weight, and increasing their PA levels. Having goals related to PA has encouraged motivation for regular PA.

### 3.5. Opportunity

Social influences (presence/lack of social support, aspiring to be a role model, time restrictions) and environmental context and resources (financial constraints, lack of accessibility) were enablers and barriers that mapped to the opportunity domain of the COM-B model.

Social support simultaneously acted both as an enabler and a barrier for preconception PA. Our quantitative findings revealed a majority of women expressed that they received support from their partners, families, and friends for their PA endeavors. We also found that family and friend support were all positively associated with higher PA levels, highlighting the importance of considering social factors in promoting PA among women. These results were supplemented by the qualitative findings, which demonstrated the unwavering support and highlighted the crucial role of partners in facilitating preconception PA.

On the contrary, the qualitative study uncovered that the absence or limitation of supportive social networks could present a substantial barrier to preconception PA. Women voiced their desire for support from friends, partners, or family members. The need to balance caregiving responsibilities with PA posed a substantial hurdle, further highlighting the intricate interplay of social support in shaping preconception PA behaviours. Furthermore, women indicated that when the responsibilities of domestic chores and caregiving are shared, time constraints lessen, allowing women to allocate more time for themselves. Women consistently face time limitations and competing priorities, including work, childcare, and household responsibilities that also interact with available social supports, particularly from partners.

The issue of time restrictions emerged as a prominent and consistent barrier to preconception PA behaviour change, as revealed by the findings from both our quantitative and qualitative studies. In the quantitative phase, 35% of women agreed with the statement that they lacked sufficient time for PA due to competing commitments, while women who reported they had enough time to participate in PA were more than twice as likely to be moderately to vigorously active.

The qualitative narratives shed light on the intricate ways in which time constraints acted as formidable barriers to engaging in regular PA. Women talked about the demands of studies or work, family obligations, and caregiving roles that made it challenging for them to allocate sufficient time, attention, and energy for regular PA amongst their countless responsibilities.

The concept of being a role model emerged as a significant enabler for preconception PA behaviour change, as evidenced by our combined quantitative and qualitative findings. In the quantitative phase, a substantial number of women expressed their agreement with the statement that they aspire to be role models for their children or future children through daily exercise. Being the role model was also found to be associated with higher levels of PA, suggesting the desire to set a positive example for children can be a powerful factor in promoting higher levels of PA, potentially leading to improved overall health for both current and future generations. Qualitative data offered deeper insights and reinforced the quantitative results. Women expressed a strong sense of consciousness regarding their position as role models, particularly in front of their children. Women also saw themselves as a role model for their families as a powerful motivator for weight loss. Women’s desire to set a positive example for their children and families served as a compelling driving force, encouraging them to adopt and sustain healthier habits during the preconception period.

The lack of accessibility to proper infrastructure was only identified in the qualitative phase of our study as a substantial barrier to preconception PA for women. Women expressed their frustrations with inadequate physical environments for PA. The suboptimal infrastructure, such as uneven or poorly maintained pathways, hindered women’s ability to engage in PA, especially when caring for children. The lack of accessible spaces and facilities not only limits the opportunities for preconception PA but also reinforces sedentary behaviours, potentially impacting overall health and well-being.

Both quantitative and qualitative findings shed light on the complex issue of affordability as a barrier to preconception PA for women. Qualitatively, financial constraints such as costs associated with gym memberships, fitness classes, and childcare presented as a significant obstacle to participation in regular PA. Women recounted their experiences of how the financial burden associated with gym memberships and childcare services can act as a significant deterrent to regular PA. However, it is important to acknowledge that perceptions of PA among women may, at times, be influenced by the misconception that it involves costly endeavors, such as gym memberships, specialized equipment, or structured fitness classes. Given the misunderstandings among women about what types of activities constitute PA, it is imperative to emphasize that affordable and accessible activities like walking can significantly contribute to overall health and well-being.

The quantitative study presented a different perspective to this discourse on the fiscal barriers to PA, with only 22% of women agreeing that they find doing regular exercise expensive. While this numerical representation suggests a lower prevalence of the perception of high cost, the qualitative findings bring to light the real-world financial constraints faced by some women. While not all women may perceive exercise as expensive, the lived experiences of some women reveal the practical challenges associated with accessing affordable fitness options.

## 4. Discussion

Our mixed-method study has shed light on the multifaceted nature of behaviour change in preconception PA among women of reproductive age. We contribute to the literature by enhancing our understanding of the factors that facilitate or impede preconception behaviour change. While we can categorize these factors into useful groups (e.g., knowledge, beliefs about consequences, goals, emotions, beliefs in capabilities, social influences, and environmental context and consequences), it is crucial to acknowledge the nuances in how and why different factors affect participation in preconception PA.

We identified awareness of the importance of preconception PA as an enabler, aligning with previous research emphasizing the role of knowledge in shaping women’s preconception behaviours [19]. Recognizing this importance can motivate behaviour change, but it is essential to note that knowledge alone is not the sole catalyst for change [43]. Conversely, misconceptions equating PA solely with vigorous exercise can act as a knowledge barrier, discouraging women from engaging in any form of PA. Clarifying the public’s understanding of what constitutes PA is crucial, emphasizing that even brief sessions of light to moderate activities offer health benefits [44].

Our findings found that leveraging the internet and social media platforms to disseminate accurate preconception PA information is vital, but the overwhelming volume of content in these spaces can lead to confusion and misinformation. This is consistent with previous studies highlighting the inconsistency and confusion of online information [45,46]. To promote informed decision-making and overcome misinformation-related barriers, interventions should empower women with evidence-based knowledge as an enabler, catalyzing the adoption of preconception PA and enhancing the health of women of reproductive age.

The belief in the benefits of preconception PA serves as an enabler for regular PA engagement. Our study found that women who strongly believe in the positive outcomes associated with preconception PA were more likely to initiate health-promoting behaviours, aligning with the health belief model [47]. This positive belief can be leveraged to change women’s attitudes, as attitudes and outcome expectations interact to shape behaviour [48]. Positive feelings associated with regular PA, as indicated by our qualitative findings, further bolster motivation for preconception PA. Experiencing joy, satisfaction, or a sense of accomplishment from regular PA can reinforce the behaviour [49]. These positive emotions can act as intrinsic motivators, encouraging women to integrate PA into their preconception lifestyle.

Lack of confidence has acted as a barrier to preconception of PA behaviour change, aligning with Bandura’s Social Cognitive Theory, which highlights the role of self-belief in behaviour change [48]. Interventions should focus on boosting women’s self-confidence via skills-building and support. Additionally, our qualitative investigation identified another barrier: women often prioritize others over themselves, placing the demands of family, work, or caregiving ahead of their own well-being. This presents a challenge to allocating time for PA, consistent with prior research indicating that women encounter significant obstacles when trying to find time for self-care and PA [50,51]. This arrangement of priorities can hinder preconception PA engagement, emphasizing the need for interventions that promote self-care and self-prioritization. Furthermore, our findings highlighted that women can only prioritize themselves when they have adequate social support in place. This aligns with previous research emphasizing the positive impact of social support on women of reproductive age [50,52]. Social support, a well-established factor in promoting health behaviours, including PA [53], provides motivation and accountability. Yet, the lack of social support is a barrier, leading to feelings of isolation and hindered motivation. Interventions can address this by fostering social connections using group activities, online communities, and support from friends and family.

The desire to be a role model emerged as an enabler, with women aspiring to set positive examples for peers, family, and future children, enhancing motivation for preconception PA. This intrinsic motivation aligns with self-determination theory, emphasizing internal sources of motivation [49]. Interventions should harness this intrinsic motivation by highlighting the potential role model status attainable through preconception PA. Leveraging this during the preconception period is crucial, as it can impact the health of future offspring. A study by Garriguet et al. established a direct relationship between parents’ PA and children’s PA, highlighting the long-term impact of parental role modelling [54]. Qualitative research further emphasized the importance of women as role models for inspiring the next generation to lead active and healthy lives [55].

Affordability and accessibility emerged as barriers in our qualitative findings. The cost of physical activities and access to fitness resources can be prohibitive, especially for those with financial constraints [56]. Interventions should prioritize offering affordable or free preconception PA options and increasing awareness of available resources. Furthermore, our study uncovered accessibility challenges, as some women lacked access to safe and suitable PA environments. Research has linked the presence of sidewalks to increased walking behaviours [57,58] and the availability of facilities like cycle paths and local parks to greater engagement in PA [59]. Addressing these challenges requires collective efforts involving not only women but also their communities, as well as higher policy engagement.

To our knowledge, this is the first study to investigate enablers and barriers influencing preconception PA for behaviour change in women of reproductive age using a mixed-method approach. The online survey provided quantitative data on the various items of the PPEBS, while the qualitative study offered an in-depth understanding of these items. The integration of both datasets resulted in a robust, comprehensive and contextually rich understanding of enablers and barriers to preconception PA. Nevertheless, it is important to note that the piloted PPEBS may have constrained the range of responses and may not have captured all the potential enablers and barriers. Thus, using a mixed-method approach allowed us to uncover additional enablers and barriers that might not have been captured in the quantitative study. We recommend future research on the PPEBS consider adding the additional enablers and barriers that were identified in the qualitative part of our study, such as enjoying doing physical activity and being too tired to engage in physical activity.

While our study provides valuable insights, it has limitations, including the sequential explanatory approach and the potential for information biases in online surveys. Nevertheless, the online survey allowed us to reach a large number of women and ensure the study had sufficient statistical power. Our sequential explanatory approach meant that we were limited in our ability to generate new and potentially unexplored enablers and barriers to preconception PA that may have been identified using other mixed methods approaches such as sequential exploratory designs. Secondly, we did not analyze variations in enablers and barriers across different socioeconomic groups or cultural perspectives because of sample size limitations, albeit this could provide additional value in future research. It is also important to acknowledge that we interviewed women only from Australia due to financial and practical considerations, which may limit the generalizability of our results to other countries.

## 5. Conclusions

Overall, this mixed-method study has provided a valuable understanding of the factors influencing preconception PA behaviours. Enablers for preconception PA included awareness of preconception PA, women’s ability to comprehend PA information available on the internet and social media platforms, belief in the benefits of preconception PA, positive emotions associated with regular PA, having goals, social support, and the desire to be a role model. Interventions should leverage these enablers for preconception PA behaviour change.

Similarly, barriers to preconception PA behaviour comprised misconceptions about the nature of PA, feeling overwhelmed by the vast amount of information available on the internet and social media, low self-confidence, low priority given to oneself, concerns about physical exertion, lack of social support, affordability issues, and limited accessibility. Interventions should focus more on debunking misconceptions surrounding PA, simplifying access to reliable information, boosting self-confidence and advocating for the prioritization of women’s own well-being. Interventions should also explore the strategies to make PA more affordable and accessible. These could take different forms to cater for the needs of women from different sociodemographic characteristics, such as digital intervention, mass media campaigns, and opportunistic intervention via health care services.

Our findings highlight the multifaceted and complex nature of enablers and barriers for preconception PA and provide a foundation for developing targeted interventions and policies aimed at promoting preconception PA among reproductive-aged women. Future research and public health efforts should consider these insights to effectively address the identified enablers and barriers for positive behaviour change in the preconception period.

## Figures and Tables

**Table 1:** Characteristics of Participants in the Quantitative Study.

Demographic Characteristic	Total Sample (*n* = 788)	Australia (*n* = 281)	US (*n* = 273)	India (*n* = 234)
Age in years (*n* = 768), *n* (%)				
18–24	141 (18.4)	14 (5.0)	58 (21.8)	69 (30.8)
25–34	313 (40.8)	113 (40.7)	102 (38.4)	98 (43.8)
35–45	314(40.9)	151 (54.3)	106 (39.9)	57 (25.5)
Marital Status, *n* (%)				
Single/Never Married	332 (42.1)	79 (28.1)	144 (52.8)	109 (46.6)
Married/De facto	416 (52.8)	194 (69.0)	99 (36.3)	123 (52.6)
Divorced/Separated/Widowed	40 (5.1)	8 (2.6)	30 (11.0)	2 (0.9)
Household Composition (*n* = 786), *n* (%)				
Couple family with children	383 (48.7)	131(46.6)	106 (39.1)	146 (62.4)
Couple family without children	133 (16.9)	71 (25.3)	43 (15.9)	19 (8.1)
Group household	113 (14.4)	31 (11.0)	42 (15.5)	40 (17.1)
One parent family	67 (8.5)	16 (5.7)	35 (12.9)	16 (6.8)
Single person household	90 (11.5)	32 (11.4)	45 (16.6)	13 (5.6)
Educational Status (*n* = 786), *n* (%)				
High School not completed	98 (12.5)	8 (2.9)	77 (28.3)	13 (5.7)
High school graduate/Diploma	100 (12.7)	25 (8.9)	61 (22.4)	14 (6.0)
Trade/Vocational/Associate degree	100 (12.7)	58 (20.7)	37 (13.6)	5 (2.1)
Bachelor’s degree	302 (38.4)	109 (38.9)	70 (25.7)	123 (52.6)
Masters and above	186 (23.7)	80 (28.6)	27 (10.0)	79 (33.8)
Paid employment, *n* (%)				
Yes	563 (71.5)	230 (81.9)	163 (59.7)	170 (72.7)
No	225 (28.6)	51 (18.2)	110 (40.3)	64 (27.4)
Pregnancy plans for future (*n* = 787), *n* (%)				
Considering in next 1 or 2 years	131 (16.7)	51 (18.2)	40 (14.7)	40 (17.1)
Considering in next 3 to 5 years	100 (12.7)	31 (11.0)	39 (14.3)	30 (12.8)
Currently trying to conceive	54 (6.9)	21 (7.5)	15 (5.5)	18 (7.7)
Have completed my family	76 (9.7)	43 (15.3)	22 (8.1)	11 (4.7)
Tried and unable to get pregnant	20 (2.5)	4 (1.4)	12 (4.4)	4 (1.7)
No plans/Not sure/Prefer not to answer	406 (51.6)	131 (46.6)	144 (52.9)	131 (56.0)
Smoking Habit (*n* = 786), *n* (%)				
Never smoked cigarettes	541 (68.8)	200 (71.2)	152 (56.1)	189 (80.8)
Currently smoking	113 (14.4)	26 (9.3)	72 (26.6)	15 (6.4)
Smoked in past	132 (16.8)	55 (19.6)	47 (17.3)	30 (12.8)
Drinking habit (*n* = 787), *n* (%)				
1–3 times a week	194 (24.7)	81 (28.8)	82 (30.2)	31 (13.3)
2–4 times a month	95 (12.1)	37 (13.2)	33 (12.1)	25 (10.7)
4 or more times a week	40 (5.1)	13 (4.6)	25 (9.2)	2 (0.9)
Monthly or less	168 (21.4)	76 (27.1)	56 (20.6)	36 (15.9)
Never	290 (36.9)	74 (26.3)	76 (27.9)	140 (59.8)
BMI (*n* = 771), *n* (%)				
Underweight	73 (9.5)	12 (4.4)	31 (11.6)	30 (13.2)
Normal	309 (40.1)	130 (47.3)	74 (27.6)	105 (46.1)
Overweight	199 (25.8)	76 (27.6)	54 (20.2)	69 (30.3)
Obesity	190 (24.6)	57 (20.7)	109 (40.7)	24 (10.5)
PA level, *n* (%)				
Low	12 (1.5)	9 (3.2)	1 (0.4)	2 (0.9)
Moderate	492 (62.4)	183 (65.1)	167 (61.2)	142 (60.7)
High	284 (36)	89 (31.7)	105 (38.5)	90 (38.5)

**Table 2:** Unadjusted Odds ratios (UOR), adjusted odds ratios (aOR), 95% Confidence Intervals (95% CIs) and *p*-values from univariable and multivariable logistic regression analyses showing associations between PA levels and PPEBS.

PPEBS	High PA Level			
	UOR (95% CI)	*p*-Value	aOR (95% CI)	*p*-Value
PA during the preconception period is important.	**1.5 (1.04–2.1)**	**0.027**	1.29 (0.86–1.95)	0.209
PA during the preconception period is important for a healthy pregnancy.	1.27 (0.89–1.82)	0.179	1.15 (0.78–1.69)	0.477
PA during the preconception period is important for a healthy baby.	1.29 (0.92–1.80)	0.145	1.06 (0.73–1.52)	0.774
I believe in the benefits of PA during the preconception period for my own general health.	1.29 (0.89–1.89)	0.181	1.09 (0.72–1.67)	0.659
I believe in the benefits of PA during the preconception period for any potential babies I have in future.	1.37 (0.97–1.95)	0.077	1.18 (0.81–1.72)	0.392
I cannot understand the PA information available on the Internet/social media related to the preconception period.	1.07 (0.76–1.52)	0.695	0.92 (0.63–1.35)	0.663
I have enough time to be physically active even though I have other commitments.	**2.43 (1.75–3.41)**	**<0.01**	**2.1 (1.47–2.99)**	**<0.01**
I do not have my partner’s support for regular PA.	**0.69 (0.45–1.04)**	**0.076**	**0.62 (0.39–0.99)**	**0.044**
I do not have my family’s support for regular PA.	**0.67 (0.45–1.04)**	**0.078**	**0.65 (0.414–1.03)**	**0.067**
I do not have my friends’ support for regular PA.	**0.65 (0.42–1.02)**	**0.064**	**0.55 (0.33–0.90)**	**0.017**
I find doing regular exercise expensive.	0.81 (0.57–1.15)	0.237	0.79 (0.53–1.17)	0.236
I want to be physically active to become a healthy person.	**1.81 (1.17–2.78)**	**0.007**	**1.63 (1.01–2.62)**	**0.045**
I want to be physically active to lose weight.	1.04 (0.76–1.50)	0.774	1.04 (0.73–1.47)	0.848
I want to be physically active to attract/maintain a partner.	1.11 (0.83–1.48)	0.503	1 (0.72–1.38)	0.99
I am physically active to improve body image.	**1.84 (1.33–2.56)**	**<0.01**	**1.59 (1.12–2.27)**	**0.01**
I am doing PA and will continue doing regular PA.	**4.09 (2.78–6.01)**	**<0.01**	**3.73(2.45–5.68)**	**<0.01**
I want to be a role model for my children/future children by exercising daily.	**2.06 (1.43–3.01)**	**<0.01**	**1.70 (1.12–2.56)**	**0.012**

Note: (1) OR based on logistic regression (2) Comparison with low/moderate PA levels (3) Model was adjusted for age, marital status, household composition, paid employment, country of residence, educational status, BMI, smoking, drinking, pregnancy planning (4) Disagree is the reference category for each statement. Significant (*p* < 0.05) findings are indicated in boldface.

**Table 3:** Demographic Characteristics of Participants for Qualitative Study.

Demographic Characteristic (*N* = 13)	Value *n* (%)
Age in years	
25–29	2 (15.4)
30–34	3 (23.1)
35–39	2 (15.4)
40–45	6 (46.2)
Ever been pregnant	
Yes	10 (76.9)
No	3 (23.1)
Planned their previous pregnancy	
Yes	9 (69.2)
No	1 (7.7)
Pregnancy plans for future	
No plans/Not sure	8 (61.5)
Currently trying to conceive	2 (15.4)
Considering in future	3 (23.1)

**Table 4:** Joint display of integrated findings from quantitative and qualitative study organized using the COM-B model.

COM-B Component	Themed groups of Enablers/Barriers	Categories of Enablers/Barriers	Joint Display of Quantitative and *Qualitative* Findings *	Integrated Summation of Findings
Capability	Knowledge	Awareness of the importance of preconception PA PA information available on social media/internet Misconceptions	Approximately three-quarters of women expressed that physical activity during the preconception period is essential for general health (78.6%), a healthy pregnancy (77.7%) and a healthy baby (74.3%). Agreement with the statement that “Physical activity during the preconception period is important” was significantly associated with higher levels of PA (univariable analysis; Table 2). *“…you know, pregnancy takes a huge toll on the body a huge toll on the body……. the healthier you are, you know, the better chance your body can, can tolerate the extra weight of the baby and, and then childbirth…”* Over three-quarters (77.6%) of women in the quantitative study reported that they can understand the PA information available on the internet or social media related to the preconception period. *“So I don’t quite know whether to trust that or not. However, it is hard to filter through what is kind of, you know, well informed and non-biased information.”* *“we when you say physical activity in [sic] the stereotypical things that come to mind of like intense exercise isn’t really something I do.”*	Awareness of the importance of preconception PA and understanding PA information available on internet/social media acted as enabler to PA. Misconceptions that only vigorous activity counts as PA and feeling overwhelmed by the information available on social media/internet acted as barriers to PA.
Motivation	Beliefs about consequences Goals Emotions Belief about capabilities	Believing in the benefits of preconception PA Positive feelings associated with regular PA Having goals Physical exhaustion and fatigue Lack of confidence No priority given to oneself	Over three-quarters of participants expressed a strong belief in the benefits of preconception PA, with 81.1% agreeing it can have a positive impact on their own general health and 75.9% perceiving benefits for the health of potential future offspring. *“Yeah, I mean, I believe, but on top of that for your future babies.”* *“What is fun, like, I love, I love movement.”* 84.7% of participants expressed a desire to be physically active to attain general health, while 69.9% aimed to lose weight, and 67.5% sought to enhance their body image. *“I would like to be super fit.”* *“So the idea now of working all day, and you’re completely exhausted. And then going actually physical exhaustion, like as I know that I need to.”* *“I think I’ve lost a lot of confidence to even just get out and do anything.”* *“There’s a kid sport something. So usually I’m running around between all of them. By the time I actually get time to sit, I want to rest, not run around or engage in exercise, so I don’t prioritise it as much as I should.”* *“I don’t go to the gym because I’ve got children. And you know, with my husband working, it’s really hard for me to find, find somewhere I can go.”* *“I don’t mean that in any sort of, I’m a supermom, or I’m a master or nothing like that. I just, yeah, I just want to try and help them succeed in the best way they can. So, where I can facilitate that I do, but it doesn’t leave a lot of time left for me.”*	Belief in the benefits of preconception PA, positive feelings associated with regular PA and having goals acted as enabler to PA. Lack of confidence, no priority given to oneself, and physical exertion acted as barriers to PA.
Opportunity	Social Influences Environmental context and resources	Presence of social support Lack of social support Aspiring to be role model Time Restrictions Financial constraints Lack of accessibility	The majority of women agreed that that they received support from their partners (84.2%), families (84.7%), and friends (86.5%). *“So, my husband built me a little gym here at the home, which is amazing. And I do that every morning.”* *“So I could like [sic] a supportive friend or partner or family member or something would be helpful.”* 75.5% of women expressed their agreement with the statement that they aspire to be role models for their children or future children through daily exercise. 35% of women agreed with the statement that they lacked sufficient time for physical activity due to competing commitments. Women who reported they had enough time to participate in PA were more than twice as likely to be moderately to vigorously active (multivariable analyses, Table 2). Only 22% of women agreed that they find doing regular exercise expensive. *“After having my second child, I did try to go to a gym, that had they advertised that they had childcare available at the gym. And I tried that. However, the tricky thing with that was that you had to book in time slots. And it was really pricey.”* *“You can imagine having a baby in a stroller, I know you’re walking down the road. And the place is just bumpy, bumpy.”*	Presence of social support and aspiring to be role model acted as enabler to PA. Time restrictions, lack of social support, financial constraints and lack of accessibility acted as barriers to PA.

* Quantitative findings are presented in normal font and qualitative findings are presented in italics.

## Data Availability

The data presented in this study are available on request from the corresponding author.

## Supplementary Materials

The following supporting information can be downloaded at: https://www.mdpi.com/article/10.3390/nu15234939/s1, Table S1: STROBE Statement—Checklist of items that should be included in reports of cross-sectional studies. Table S2: Final 17-item version of PPEBS. Table S3: Percentage distribution of participants who agreed with PPEBS statements by country. Table S4: Interview Guide for qualitative study. Figure S1: Horizontal bar charts for each statement for barriers and enablers with all response categories.
