# Supplementary material for: Enablers and Barriers Related to Preconception Physical Activity: Insights from Women of Reproductive Age Using Mixed Methods
Source: Nutrients. 2023 Nov 28;15(23):4939. doi: 10.3390/nu15234939 (PMC10707946; doi:10.3390/nu15234939)

**Supplementary Materials**

**Enablers and Barriers Related to Preconception Physical Activity: Insights from Women of Reproductive Age Using Mixed Methods**

Pragya Kandel^1^, Siew Lim^2^, Michelle Dever^1^, Prabhat Lamichhane^3^, Helen Skouteris^1,4^ Sinead Currie^5^, Briony Hill^1*^

^1^ Health and Social Care Unit, Monash University, Melbourne VIC 3004, Australia; [pragya.kandel1@monash.edu](mailto:pragya.kandel1@monash.edu)

^2^ Health Systems and Equity, Eastern Health Clinical School, Monash University, Melbourne VIC 3004, Australia; [siew.lim1@monash.edu](mailto:siew.lim1@monash.edu)

^1^ Health and Social Care Unit, Monash University, Melbourne VIC 3004, Australia; [michelle.dever@monash.edu](mailto:michelle.dever@monash.edu)

^3^ Department of Public Health, School of Psychology and Public Health, La Trobe University VIC 3086, Australia; [p.lamichhane@latrobe.edu.au](mailto:p.lamichhane@latrobe.edu.au)

^1, 4^ Warwick Business School, University of Warwick, Coventry, CV4 7AL UK; [helen.skouteris@monash.edu](mailto:helen.skouteris@monash.edu)

^5^ Division of Psychology, University of Stirling, Stirling, FK9 4LA, UK; [sinead.currie@stir.ac.uk](mailto:sinead.currie@stir.ac.uk)

^1^* Health and Social Care Unit, Monash University, Melbourne VIC 3004, Australia; [briony.hill@monash.edu](mailto:briony.hill@monash.edu)

*Author to whom correspondence should be addressed.

Table S1: STROBE Statement - Checklist of items that should be included in reports of ***cross-sectional studies***

|  | Item No | Recommendation | Page No |
| --- | --- | --- | --- |
| Title and abstract | 1 | (*a*) Indicate the study’s design with a commonly used term in the title or the abstract | 1 |
|  |  | (*b*) Provide in the abstract an informative and balanced summary of what was done and what was found | 1 |
| Introduction | | |  |
| Background/rationale | 2 | Explain the scientific background and rationale for the investigation being reported | 1, 2 |
| Objectives | 3 | State specific objectives, including any prespecified hypotheses | 2 |
| Methods | | |  |
| Study design | 4 | Present key elements of study design early in the paper | 3 |
| Setting | 5 | Describe the setting, locations, and relevant dates, including periods of recruitment, exposure, follow-up, and data collection | 3, 4 |
| Participants | 6 | (*a*) Give the eligibility criteria, and the sources and methods of selection of participants | 3 |
| Variables | 7 | Clearly define all outcomes, exposures, predictors, potential confounders, and effect modifiers. Give diagnostic criteria, if applicable | 3,4 |
| Data sources/ measurement | 8* | For each variable of interest, give sources of data and details of methods of assessment (measurement). Describe comparability of assessment methods if there is more than one group | 3,4 |
| Bias | 9 | Describe any efforts to address potential sources of bias | N/A |
| Study size | 10 | Explain how the study size was arrived at | 4 |
| Quantitative variables | 11 | Explain how quantitative variables were handled in the analyses. If applicable, describe which groupings were chosen and why | 4,5 |
| Statistical methods | 12 | (*a*) Describe all statistical methods, including those used to control for confounding | 4,5 |
|  |  | (*b*) Describe any methods used to examine subgroups and interactions | N/A |
|  |  | (*c*) Explain how missing data were addressed | N/A |
|  |  | (*d*) If applicable, describe analytical methods taking account of sampling strategy | N/A |
|  |  | (*e*) Describe any sensitivity analyses | N/A |
| Results | | |  |
| Participants | 13* | (a) Report numbers of individuals at each stage of study—eg numbers potentially eligible, examined for eligibility, confirmed eligible, included in the study, completing follow-up, and analysed | 4 |
|  |  | (b) Give reasons for non-participation at each stage | N/A |
|  |  | (c) Consider use of a flow diagram | 4 |
| Descriptive data | 14* | (a) Give characteristics of study participants (eg demographic, clinical, social) and information on exposures and potential confounders | 6 |
|  |  | (b) Indicate number of participants with missing data for each variable of interest | 6,7,8 |
| Outcome data | 15* | Report numbers of outcome events or summary measures | 7,8, Fig S1 |
| Main results | 16 | (*a*) Give unadjusted estimates and, if applicable, confounder-adjusted estimates and their precision (eg, 95% confidence interval). Make clear which confounders were adjusted for and why they were included | 8 |
|  |  | (*b*) Report category boundaries when continuous variables were categorized | N/A |
|  |  | (*c*) If relevant, consider translating estimates of relative risk into absolute risk for a meaningful time period | N/A |
| Other analyses | 17 | Report other analyses done—eg analyses of subgroups and interactions, and sensitivity analyses | N/A |
| Discussion | | |  |
| Key results | 18 | Summarise key results with reference to study objectives | 7 |
| Limitations | 19 | Discuss limitations of the study, taking into account sources of potential bias or imprecision. Discuss both direction and magnitude of any potential bias | 16 |
| Interpretation | 20 | Give a cautious overall interpretation of results considering objectives, limitations, multiplicity of analyses, results from similar studies, and other relevant evidence | 16,17 |
| Generalisability | 21 | Discuss the generalisability (external validity) of the study results | N/A |
| Other information | | |  |
| Funding | 22 | Give the source of funding and the role of the funders for the present study and, if applicable, for the original study on which the present article is based | 17 |

*Give information separately for exposed and unexposed groups.

**Note:** An Explanation and Elaboration article discusses each checklist item and gives methodological background and published examples of transparent reporting. The STROBE checklist is best used in conjunction with this article (freely available on the Web sites of PLoS Medicine at http://www.plosmedicine.org/, Annals of Internal Medicine at http://www.annals.org/, and Epidemiology at http://www.epidem.com/). Information on the STROBE Initiative is available at www.strobe-statement.org.

Table S2: Final 17-item version of PPEBS

| **Item** | **PPEBS** |
| --- | --- |
| 1 | Physical activity during the preconception period is important. |
| 2 | Physical activity during the preconception period is important for healthy pregnancy. |
| 3 | Physical activity during the preconception period is important for healthy baby. |
| 4 | I believe in the benefits of physical activity during the preconception period for my own general health. |
| 5 | I believe in the benefits of physical activity during the preconception period for any potential babies I have in future. |
| 6 | I cannot understand the physical activity information available on the Internet/social media related to the preconception period. |
| 7 | I have enough time to be physically active even though I have other commitments. |
| 8 | I don’t have my partner’s support for regular physical activity. |
| 9 | I don’t have my family’s support for regular physical activity. |
| 10 | I don’t have my friends’ support for regular physical activity. |
| 11 | I find doing regular exercise expensive. |
| 12 | I want to be physically active to become a healthy person. |
| 13 | I want to be physically active to lose weight. |
| 14 | I want to be physically active to attract/maintain a partner. |
| 15 | I am physically active to improve body image. |
| 16 | I am doing physical activity and will continue doing regular physical activity. |
| 17 | I want to be a role model for my children/future children by exercising daily. |

Table S3: Percentage distribution of participants who agreed with PPEBS statements by country

| **PPEBS** | **Australia** | | **US** | | **India** | |
| --- | --- | --- | --- | --- | --- | --- |
|  | **Agree %** | **n** | **Agree %** | **n** | **Agree %** | **n** |
| Physical activity during the preconception period is important. | 83.6 | 281 | 72.9 | 273 | 77.4 | 234 |
| Physical activity during the preconception period is important for healthy pregnancy. | 82.1 | 280 | 71.4 | 273 | 78.2 | 234 |
| Physical activity during the preconception period is important for healthy baby. | 74.0 | 281 | 72.8 | 272 | 75.6 | 234 |
| I believe in the benefits of physical activity during the preconception period for my own general health. | 82.9 | 281 | 75.5 | 273 | 83.8 | 234 |
| I believe in the benefits of physical activity during the preconception period for any potential babies I have in future. | 78.6 | 280 | 72.2 | 273 | 77.4 | 234 |
| I cannot understand the physical activity information available on the Internet/social media related to the preconception period. | 17.8 | 281 | 25.3 | 273 | 24.8 | 234 |
| I have enough time to be physically active even though I have other commitments. | 60.9 | 281 | 68.5 | 273 | 68.4 | 234 |
| I don’t have my partner’s support for regular physical activity. | 11.0 | 281 | 20.9 | 273 | 16.7 | 233 |
| I don’t have my family’s support for regular physical activity. | 11.4 | 281 | 19.8 | 273 | 16.2 | 234 |
| I don’t have my friends’ support for regular physical activity. | 8.9 | 280 | 16.1 | 273 | 16.2 | 234 |
| I find doing regular exercise expensive. | 20.7 | 280 | 22.0 | 273 | 25.2 | 234 |
| I want to be physically active to become a healthy person. | 85.8 | 281 | 76.9 | 273 | 90.2 | 234 |
| I want to be physically active to lose weight. | 70.1 | 281 | 71.1 | 273 | 68.0 | 234 |
| I want to be physically active to attract/maintain a partner. | 40.7 | 280 | 47.3 | 273 | 53.7 | 233 |
| I am physically active to improve body image. | 64.1 | 281 | 64.1 | 273 | 76.9 | 234 |
| I am doing physical activity and will continue doing regular physical activity. | 69.0 | 281 | 60.4 | 273 | 83.8 | 234 |
| I want to be a role model for my children/future children by exercising daily. | 70.7 | 280 | 71.1 | 273 | 87.6 | 234 |

Table S4: Interview Guide for qualitative study

| **Category** | **Interview Questions** |
| --- | --- |
| Consent | Even though you have already given consent to participate and record this interview, I could like to ask again. After reading the explanatory statement that we sent in the email, do you consent to participate in this research? (Yes/No)  When I press record button from my side, you will get a small box asking about the recording which you will have to press yes. |
| Introductory question | Thank you so much for joining in. Please let me know if you need break anytime or if there is anything you don’t understand during the interview.  Could you please tell me about yourself? |
| Definition for preconception | In our interview today, we are going to talk about the preconception physical activity. For the purpose of our research, we have defined preconception as the time before conception – Time before women gets pregnant including the time even when she is not planning pregnancy  So, during the interview, whenever I say physical activity, I will be talking about the physical activity before conception. |
| Main questions | Let’s start by talking about PA habits. Can you share some of your thoughts on PA habits?  *(Prompts: How important do you think PA is important for child/healthy pregnancy?* |
|  | Can you we can talk about the benefits of PA? What do you think are the benefits about the PA?  *(Prompts: health outcomes, e.g. for child)*  *Do you believe in benefits of physical activity for your general health/babies?* |
|  | Many people turn to the internet for information about preconception physical activity. What do you think about the information on the internet about preconception physical activity?  *(Prompts: Do you understand the content?)* |
|  | Please tell me about the things that motivate you to be physically active. |
|  | Please tell me about your goals for physical activity?  *(Prompts: become a healthy person, lose weight, attract/maintain a partner, role model for children/ future children)* |
|  | What barriers are for you to be physically active?  *(Prompts: time, support from partner/family/friends, expensive, accessibility, is there a particular barrier that stands out to you? Can you tell me more about it? How is this barrier for you?)* |
| Concluding question | Today we talked about PA. Is there anything else that you would like to add? Do you have any questions for me? |
| Demographic questions | Thank you so much for your thoughts.  Before ending questions, I could like to ask about some questions about yourself to understand our participant groups.  Do you mind sharing your age?  *This next question might too sensitive for some so please free to decline to answer.*  Have you ever been pregnant? If yes, was the pregnancy planned?  What are your current plans about getting pregnant?  Would like to have an executive summary of this research sent to you when we have finished? |

**Figure S1:** Horizontal bar charts for each statement for barriers and enablers with all response categories


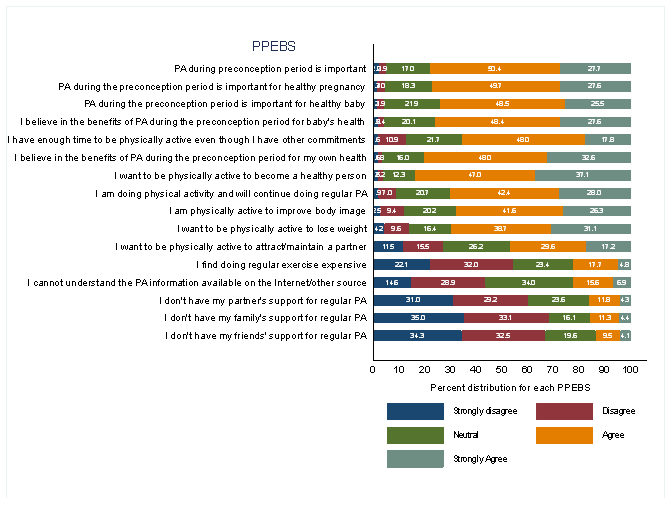

Supplement: Supplementary file 1 [file nutrients-15-04939-s001.zip › nutrients-2716668-supplementary.docx]
